# Antibiotic resistance among bacterial conjunctival pathogens collected in the Antibiotic Resistance Monitoring in Ocular Microorganisms (ARMOR) surveillance study

**DOI:** 10.1371/journal.pone.0205814

**Published:** 2018-10-18

**Authors:** Penny A. Asbell, Heleen H. DeCory

**Affiliations:** 1 Icahn School of Medicine at Mount Sinai, New York, New York, United States of America; 2 Bausch + Lomb, Rochester, New York, United States of America; Seconda Universita degli Studi di Napoli, ITALY

## Abstract

The Antibiotic Resistance Monitoring in Ocular Microorganisms (ARMOR) surveillance study evaluates in vitro antibiotic resistance among *Staphylococcus aureus*, coagulase-negative staphylococci (CoNS), *Streptococcus pneumoniae*, *Pseudomonas aeruginosa*, and *Haemophilus influenzae* isolates from ocular infections. Here we report resistance rates and trends among conjunctival-sourced ocular isolates collected across the US from 2009 through 2016. A total of 1198 conjunctival isolates (483 *S*. *aureus*, 305 CoNS, 208 *H*. *influenzae*, 118 *S*. *pneumoniae*, and 84 *P*. *aeruginosa)* were collected from patients with presumed bacterial conjunctivitis from 57 sites across 40 states. A large proportion of staphylococci demonstrated resistance to oxacillin and azithromycin, while resistance was low against the majority of antibiotics tested for *S*. *pneumoniae*, *P*. *aeruginosa*, and *H*. *influenzae*. Multidrug resistance (≥3 antibiotic classes) was found in 30.2% of *S*. *aureus* and 39.0% of CoNS isolates, and methicillin resistance more than doubled the rate of multi-drug resistance (methicillin-resistant *S*. *aureus* [MRSA], 76.5%; methicillin-resistant CoNS isolates, 72.8%). There was a pattern of increasing mean percent resistance with increasing age by decade of life among *S*. *aureus*, MRSA, and CoNS (*P*≤0.038). Over the eight-year study period, there were small yet significant decreases in resistance rates among *S*. *aureus* to azithromycin, ciprofloxacin, tobramycin, trimethoprim, and oxacillin (*P≤*0.003), and among CoNS and *P*. *aeruginosa* (both *P*<0.05) to ciprofloxacin. These data indicate that antibiotic resistance is high, but did not increase, among conjunctival-sourced isolates collected in the US from 2009 through 2016. For certain antibiotic/pathogen combinations, there was a trend of decreased resistance, including a decrease in oxacillin resistance among *S*. *aureus*.

## Introduction

Conjunctivitis is a common ocular infection affecting all age groups [1]. While a viral etiology is responsible for the majority of adult cases, bacterial conjunctivitis is the second most common cause in adults and may be the primary cause in children [2,3]. Causative bacterial agents among adults are most frequently staphylococcal species, followed by *Streptococcus pneumoniae* and *Haemophilus influenzae*, with *Pseudomonas aeruginosa* common in contact lens wearers [4–6]. In children, *H*. *influenzae* is the most common pathogen, followed by *S*. *pneumoniae*, *Staphylococcus aureus*, and *Staphylococcus epidermidis* [7–10].

While bacterial conjunctivitis is generally self-limiting, treatment with topical antibiotics is associated with earlier clinical and microbiological remission, as well as decreased discomfort and morbidity [1,9,11,12]. In children, treatment is especially important as many US state departments of health require that children be kept home from day care/school until they are asymptomatic or under treatment [13]. Antibiotic therapy is typically initiated empirically, with guidelines recommending cultures only in severe, chronic, recurrent, or treatment-unresponsive cases [1].

Since the introduction of antibiotics, bacterial resistance has continued to pose an ongoing problem across infectious diseases, and ocular infection pathogens are no exception [4,14–25]. The presence of antibiotic resistance among ocular pathogens is of concern, as it complicates the choice of antibiotic and may lead to treatment failure [26–30]. However, few surveillance studies have specifically focused on susceptibility patterns among ocular pathogens, and most have been single-center studies [4,20,23,26,31].

To date, there have been only two nationwide, multi-center prospective surveillance studies, one of which is currently active. The Ocular Tracking Resistance in US Today (TRUST) study which evaluated ocular pathogens collected from 2005 through 2008 reported in vitro resistance among *S*. *aureus*, *H*. *influenzae*, and *S*. *pneumoniae* isolates to a number of commonly used topical antibiotics, with in vitro methicillin resistance increasing significantly among *S*. *aureus* and CoNS over the three-year study period [32–34]. The Antibiotic Resistance Monitoring in Ocular Microorganisms (ARMOR) study, initiated in January 2009, is the only surveillance initiative specific to ocular pathogens currently ongoing. Comprehensive five- and seven-year cumulative findings of the ARMOR study have been published [15,34]. Herein, we report in vitro antibiotic resistance profiles and trends for 1198 bacterial isolates obtained from patients with presumed bacterial conjunctivitis over 8 years of the ARMOR surveillance study.

## Methods

Isolates of *S*. *aureus*, CoNS, *S*. *pneumoniae*, *H*. *influenzae*, and *P*. *aeruginosa* cultured from eye infections were submitted by participating US sites as part of the ARMOR surveillance study [15,16,34]. From 2009–2013, each participating site was invited to submit up to 65 ocular isolates per collection year, including no more than 20 *S*. *aureus*, 20 CoNS, 5 *S*. *pneumoniae*, 5 *H*. *influenzae*, and 15 *P*. *aeruginosa*; whereas from 2014–2016, participating sites were invited to submit a maximum of 50 isolates per collection year of *S*. *aureus*, CoNS, *S*. *pneumoniae*, *H*. *influenzae*, and *P*. *aeruginosa*, with no more than 12 isolates of any given species. The central laboratory in ARMOR obtained pure subcultures of bacterial isolates from each of the 87 participating clinical sites from 2009–2016; not all sites submitted samples throughout all 8 years. The current study reports antibiotic resistance rates and trends among ocular isolates collected from the conjunctiva from January 1, 2009, through December 31, 2016.

There were no human participants involved in ARMOR, or specimens or tissue samples actively taken as part of ARMOR. Because this was a laboratory study, institutional review board approval was left to the discretion of participating sites, but not required because no patient identifying information was provided with isolates. The present analyses were limited to isolates characterized by participating centers as originating from the conjunctiva.

Species confirmation and antibiotic resistance profile determination for each isolate were performed at a central laboratory (Eurofins Medinet, Chantilly, VA [2009–2013]; IHMA Inc, Schaumburg, IL [2014–2016]). Minimum inhibitory concentrations (MIC) were determined by broth microdilution according to the Clinical and Laboratory Standards Institute (CLSI) methods using frozen antimicrobial microtiter panels [35–37]. Each isolate was tested against antibiotics from 10 different classes as appropriate based on species. Antibiotics included the fluoroquinolones (ciprofloxacin, moxifloxacin, gatifloxacin, besifloxacin, levofloxacin, and ofloxacin), macrolides (azithromycin), aminoglycosides (tobramycin), lincosamides (clindamycin), penicillins (oxacillin and/or penicillin), dihydrofolate reductase inhibitors (trimethoprim), polypeptides (polymyxin B), amphenicols (chloramphenicol), tetracyclines (tetracycline) and glycopeptides (vancomycin). Not all antibiotic classes were tested in each of the 8 years of the study period.

CLSI interpretive criteria [38–45], which are based on data from systemic infections, were used to interpret MICs as susceptible, intermediate, or resistant for each species/antibiotic combination (when available). Isolates were reported as resistant if they classified as either intermediate or resistant during MIC testing. For staphylococci isolates, susceptibility to oxacillin was used to categorize isolates as methicillin-resistant (MR) or methicillin-susceptible (MS). Susceptibility of *S*. *pneumoniae* isolates to penicillin was based on the breakpoint for oral penicillin. Resistance to three or more classes of antibiotics was defined as multidrug resistance.

For analysis by age of the source patient, isolates were categorized into age groups by decade of life. For analysis by geography, isolates were categorized into four regions based on state in which the participating center was located: Western US (AK, AZ, CA, CO, HI, ID, MT, NV, NM, OR, UT, WA, WY), Midwestern US (IA, IL, IN, KS, KY, MI, MN, MO, ND, NE, OH, SD, WI), Southern US (AL, AR, FL, GA, LA, MD, MS, NC, OK, SC, TN, TX, VA, WV) and Northeastern US (CT, DE, MA, ME, NH, NJ, NY, PA, RI, VT).

To determine if resistance among conjunctiva isolates differed across age groups or geographic location, a one-way analysis of variance (ANOVA) was conducted. Because not all antibiotic classes were tested in each of the 8 years of the study period, the ANOVA utilized the means of the percentage of drug classes to which each isolate of a species/species group was resistant. Results of significance were then subjected to further testing using Tukey’s Honestly Significant Differences (HSD) All-Pairwise Comparisons Test [46], which used the *P*<0.1 criterion for statistical significance.

Where indicated, differences among staphylococcal isolates based on MR status were determined using a Chi-Square Test followed by a multiple comparisons test for proportions. The Cochran-Armitage test for linear trends in a proportion [47] was used to evaluate changes in resistance rates over time. Statistical significance was defined as a *P* value <0.05 unless otherwise indicated. Statistical testing was performed using Statistix 10 (Analytical Software, Tallahassee, FL) or GraphPad Prism 5.01 (San Diego, CA).

## Results

**Fig 1:**
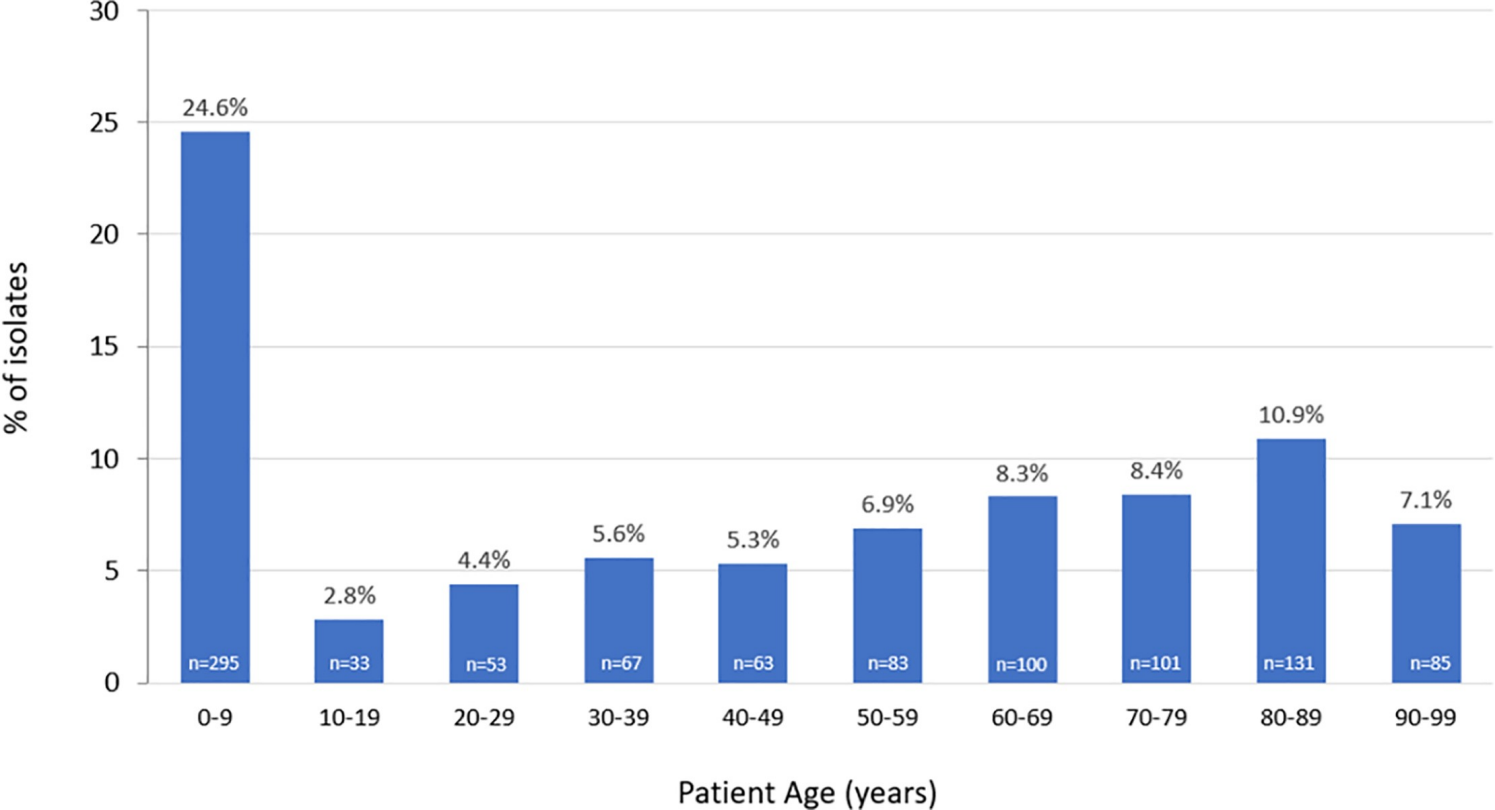
Proportion of conjunctival isolates collected in the ARMOR study, stratified by patient age. Missing: n = 187 (15.6%).

A total of 1198 isolates (483 *S*. *aureus*, 305 CoNS, 208 *H*. *influenzae*, 118 *S*. *pneumoniae*, and 84 *P*. *aeurginosa)* from the conjunctiva were collected from 57 participating centers across 40 states in the US. The majority of isolates came from the Midwest region (n = 499; 41.7%), followed by the Northeast (n = 286; 23.9%), West (n = 280; 23.4%), and South (n = 133; 11.1%). An equal percentage of isolates were collected from male (n = 558; 46.6%) and female (n = 560; 46.7%) patients (unknown for 80 [6.7%] isolates). One-quarter (24.6%) of the conjunctival-sourced isolates were obtained from patients aged 0–9 years (**Fig 1**).

### In vitro antibiotic resistance rates

**Table 1** presents MIC_90_ (the MIC required to inhibit the growth of 90% of isolates) and antibiotic resistance profiles for the *S*. *aureus*, CoNS, *S*. *pneumoniae*, and *P*. *aeruginosa* isolates collected from presumed conjunctivitis cases.

**Table 1:** Minimum inhibitory concentrations and resistance profiles for conjunctival *S*. *aureus*, coagulase-negative staphylococci, *S*. *pneumoniae*, and *P*. *aeruginosa* from ARMOR 2009–2016.

Organism(s) Antibiotic	N^o^	MIC_90_ (μg/mL)	Susceptible	Intermediate	Resistant
			%	%	%
***S*.* aureus***					
Ofloxacin	(All) 414	>8	66.7	0.72	32.6
	(MSSA) 288	8	86.5	1.0	12.5
	(MRSA) 126	64	21.4	0.0	78.6
Ciprofloxacin	(All) 483	128	66.5	1.0	32.5
	(MSSA) 330	8	87.0	1.2	11.8
	(MRSA) 153	256	22.2	0.7	77.1
Levofloxacin	(All) 414	32	67.6	1.9	30.4
	(MSSA) 288	4	87.9	1.4	10.8
	(MRSA) 126	128	21.4	3.2	75.4
Gatifloxacin	(All) 414	8	67.4	3.4	29.2
	(MSSA) 288	2	87.5	2.4	10.1
	(MRSA) 126	16	21.4	5.6	73.0
Moxifloxacin	(All) 483	8	68.3	6.0	25.7
	(MSSA) 330	1	89.1	3.3	7.6
	(MRSA) 153	16	23.5	11.8	64.7
Besifloxacin	(All) 483	1	na	na	na
	(MSSA) 330	0.25	na	na	na
	(MRSA) 153	4	na	na	na
Azithromycin	(All) 483	>512	43.3	0.4	56.3
	(MSSA) 330	>512	60.3	0.6	39.1
	(MRSA) 153	>512	6.5	0.0	93.5
Clindamycin	(All) 483	>2	84.7	1.2	14.1
	(MSSA) 330	0.25	92.4	1.5	6.1
	(MRSA) 153	>16	68.0	0.7	31.4
Chloramphenicol	(All) 414	8	94.2	5.1	0.7
	(MSSA) 288	8	96.2	3.1	0.7
	(MRSA) 126	16	89.7	9.5	0.8
Tobramycin	(All) 483	128	81.0	2.3	16.8
	(MSSA) 330	1	95.2	0.3	4.6
	(MRSA) 153	256	50.3	6.5	43.1
Trimethoprim	(All) 414	4	94.2	0.0	5.8
	(MSSA) 288	4	96.2	0.0	3.8
	(MRSA) 126	>128	89.7	0.0	10.3
Vancomycin	(All) 483	1	100	0.0	0.0
	(MSSA) 330	1	100	0.0	0.0
	(MRSA) 153	1	100	0.0	0.0
**Coagulase-Negative Staphylococci**^a^					
	(All) 260	>8	71.5	0.0	28.5
Ofloxacin	(MSCoNS) 137	1	90.5	0.0	9.5
	(MRCoNS) 123	32	50.4	0.0	49.6
Ciprofloxacin	(All) 305	64	69.5	2.0	28.5
	(MSCoNS) 158	4	89.2	0.6	10.1
	(MRCoNS) 147	64	48.3	3.4	48.3
Levofloxacin	(All) 260	16	71.5	3.5	25.0
	(MSCoNS) 137	0.5	90.5	0.7	8.8
	(MRCoNS) 123	128	50.4	6.5	43.1
Gatifloxacin	(All) 260	4	71.5	4.6	23.9
	(MSCoNS) 137	0.5	90.5	2.2	7.3
	(MRCoNS) 123	32	50.4	7.3	42.3
Moxifloxacin	(All) 305	8	72.8	4.3	23.0
	(MSCoNS) 158	1	89.9	2.5	7.6
	(MRCoNS) 147	32	54.4	6.1	39.5
Besifloxacin	(All) 305	1	na	na	na
	(MSCoNS) 158	0.25	na	na	na
	(MRCoNS) 147	2	na	na	na
Azithromycin	(All) 305	>512	36.7	0.0	63.3
	(MSCoNS) 158	>512	53.2	0.0	46.8
	(MRCoNS) 147	>512	19.1	0.0	81.0
Clindamycin	(All) 305	>64	68.9	5.6	25.6
	(MSCoNS) 158	16	79.8	7.0	13.3
	(MRCoNS) 147	>64	57.1	4.1	38.8
Chloramphenicol	(All) 260	8	98.1	0.8	1.2
	(MSCoNS) 137	4	97.8	0.7	1.5
	(MRCoNS) 123	8	98.4	0.8	0.8
Tobramycin	(All) 305	8	83.9	6.6	9.5
	(MSCoNS) 158	4	94.3	3.8	1.9
	(MRCoNS) 147	32	72.8	9.5	17.7
Trimethoprim	(All) 260	256	71.9	0.0	28.1
	(MSCoNS) 137	>128	83.9	0.0	16.1
	(MRCoNS) 123	>256	58.5	0.0	41.5
Vancomycin	(All) 305	2	100	0.0	0.0
	(MSCoNS) 158	2	100	0.0	0.0
	(MRCoNS) 147	2	100	0.0	0.0
***S*.* pneumoniae***					
Ofloxacin	96	2	100	0.0	0.0
Ciprofloxacin	118	1	na	na	na
Levofloxacin	96	1	100	0.0	0.0
Gatifloxacin	96	0.25	100	0.0	0.0
Moxifloxacin	118	0.25	100	0.0	0.0
Besifloxacin	118	0.06	na	na	na
Azithromycin	118	>128	68.6	0.0	31.4
Chloramphenicol	118	4	98.3	0.0	1.7
Ceftriaxone	79	1	94.9	5.1	0.0
Imipenem	57	0.12	91.2	5.3	3.5
Penicillin	118	1	70.3	20.3	9.3
Tetracycline	39	>4	84.6	0	15.4
***P*.* aeruginosa***					
Ofloxacin	71	2	91.6	1.4	7.0
Ciprofloxacin	84	1	91.7	1.2	7.1
Levofloxacin	71	1	93.0	2.8	4.2
Gatifloxacin	71	1	91.6	2.8	5.6
Moxifloxacin	84	2	na	na	na
Besifloxacin	84	4	na	na	na
Tobramycin	84	1	96.4	1.2	2.4
Imipenem	48	8	85.4	4.2	10.4
Polymyxin B	71	2	95.8	2.8	1.4

^a^ The CoNS isolates included *Staphylococcus capitis* (n = 9), *Staphylococcus caprae* (n = 1), *Staphylococcus epidermidis* (n = 232), *Staphylococcus haemolyticus* (n = 9), *Staphylococcus hominis* (n = 13), *Staphylococcus lugdunensis* (n = 8), *Staphylococcus pasteuri* (n = 2), *Staphylococcus pettenkoferi* (n = 1), *Staphylococcus simulans* (n = 1), *Staphylococcus warneri* (n = 10), and unspeciated CoNS (n = 19).

Abbreviations: CoNS, coagulase-negative staphylococci; MIC_90_, minimum inhibitory concentration that inhibits the growth of 90% of indicated isolates; MRCoNS, methicillin-resistant CoNS; MRSA, methicillin-resistant *S*. *aureus*; MSCoNS, methicillin-susceptible CoNS; MSSA, methicillin-susceptible *S*. *aureus*; na, Clinical and Laboratory Standards Institute interpretive breakpoints currently not available/applicable.

Percent susceptible, intermediate and resistant may not add to 100.0 due to rounding.

Of *S*. *aureus* isolates, cumulative in vitro resistance to azithromycin, ciprofloxacin, tobramycin and oxacillin (MR *S*. *aureus* [MRSA]) was 56.7%, 33.6%, 19.1%, and 31.7%, respectively. Resistance to chloramphenicol (5.8%) and trimethoprim (5.8%) was low. Compared to MS *S*. *aureus* (MSSA), MRSA strains demonstrated greater in vitro resistance to azithromycin (93.5% vs 39.7%), tobramycin (49.7% vs 4.9%), and the fluoroquinolones ciprofloxacin, levofloxacin, gatifloxacin, and moxifloxacin (75.6%-78.6% vs 10.9%-13.0%). MIC_90_s were lower for newer fluoroquinolones (besifloxacin, moxifloxacin, gatifloxacin) as compared to older fluoroquinolones (ofloxacin, ciprofloxacin, and levofloxacin). The lowest MIC_90_s among all tested antibiotics were for besifloxacin (MSSA, 0.25 μg/mL; MRSA, 4 μg/mL) and vancomycin (MSSA and MRSA, both 1 μg/mL).

Among CoNS isolates, cumulative in vitro resistance was greatest to azithromycin (63.3%) and oxacillin (MRCoNS; 48.2%), followed by ciprofloxacin (30.5%), trimethoprim (28.1%), and tobramycin (16.1%). Resistance to chloramphenicol was low (1.9%). As observed with MRSA, higher rates of resistance were found among MRCoNS isolates when compared to MSCoNS. As observed with *S*. *aureus*, MIC_90_s for both MSCoNS and MRCoNS were lower with newer fluoroquinolones as compared with older fluoroquinolones, and besifloxacin exhibited the lowest MIC_90_ (MSCoNS, 0.25 μg/mL; MRCoNS, 2 μg/mL).

In vitro resistance among *S*. *pneumoniae* isolates was low for the majority of antibiotics tested, and all isolates were susceptible to tested fluoroquinolones (**Table 1**). However, resistance to azithromycin (31.4%), tetracycline (15.4%), imipenem (8.8%) and penicillin (29.7%) was noted. Besifloxacin demonstrated the lowest MIC_90_ of all tested antibiotics (0.06 μg/mL).

The *P*. *aeruginosa* isolates demonstrated high rates of in vitro susceptibility to tested antibiotics, with the highest resistance rates being 14.6% for imipenem, 8.5% for gatifloxacin, 8.5% for ofloxacin, and 8.3% for ciprofloxacin. MIC_90_s were lowest for ciprofloxacin, gatifloxacin, levofloxacin, and tobramycin (1 μg/mL for all).

Among *H*. *influenzae* isolates, in vitro resistance was observed in only 4 isolates (2 –azithromycin; 1 –tetracycline/chloramphenicol; 1 –tetracycline). All other isolates were susceptible to all antibiotics tested. For the fluoroquinolones tested, MIC_90_s for besifloxacin, moxifloxacin, and ofloxacin were each 0.03 μg/mL and 0.015 μg/mL for ciprofloxacin, gatifloxacin, and levofloxacin. Other MIC_90_s included 2 μg/mL for azithromycin, 1 μg/mL for chloramphenicol, and 0.5 μg/mL for tetracycline.

**Fig 2:**
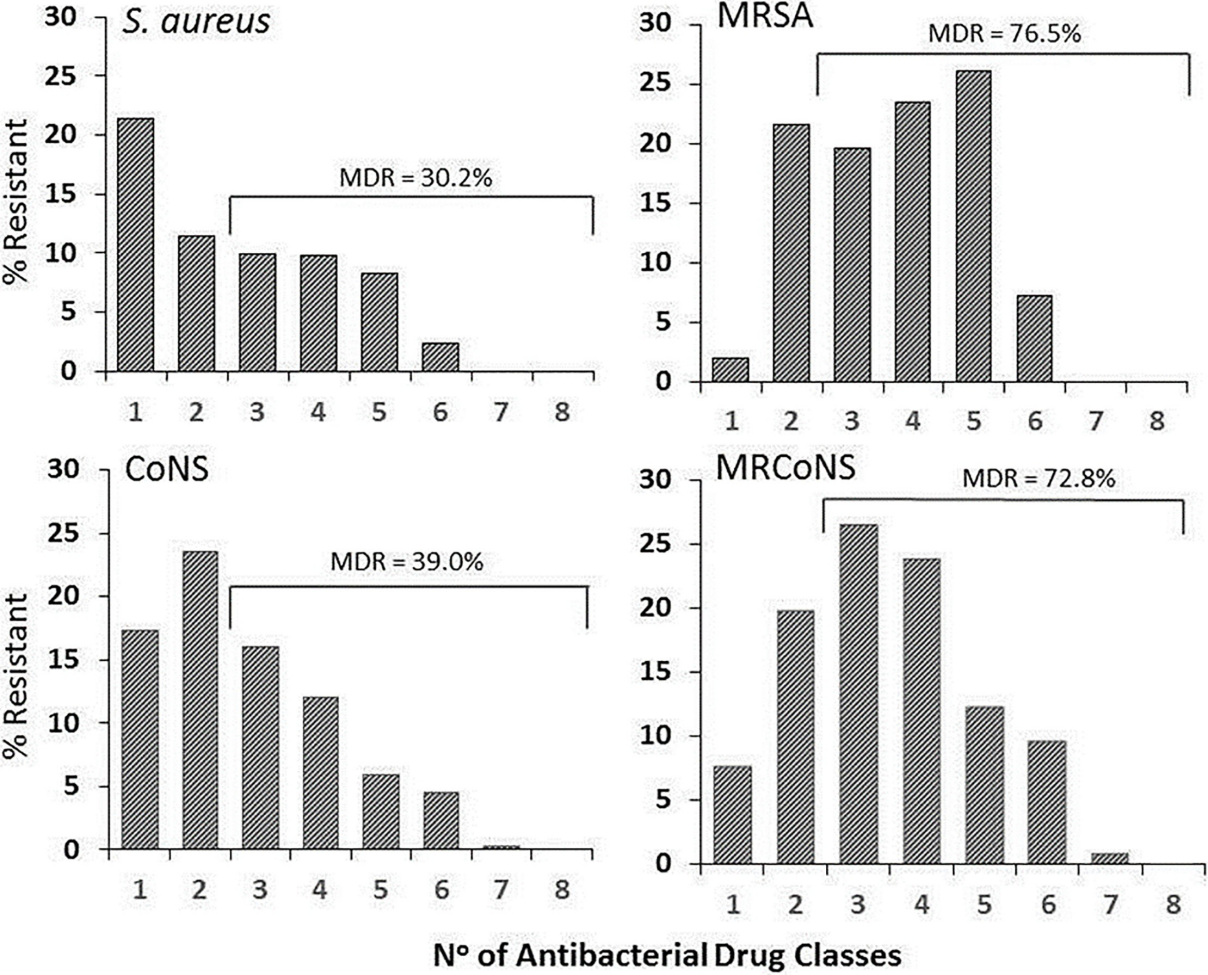
Multidrug resistance (MDR)^a^ among conjunctival *S*. *aureus* and coagulase-negative staphylococci. Isolates were tested against ciprofloxacin, azithromycin, clindamycin, chloramphenicol, tobramycin, oxacillin, tetracycline, vancomycin, and trimethoprim. Percent resistance includes intermediate resistance. ^a^ Multidrug resistance defined as resistance to 3 or more classes of antibiotics. Abbreviations: MRSA, methicillin-resistant *S*. *aureus*; CoNS, coagulase-negative staphylococci; MRCoNS, methicillin-resistant coagulase-negative staphylococci.

Multidrug in vitro resistance (resistance to ≥3 classes of antibiotics) was found in 30.2% of *S*. *aureus* and 39.0% of CoNS isolates (**Fig 2)**. These percentages increased to 76.5% and 72.8% when examining only MRSA and MRCoNS, respectively. In contrast, MS staphylococcal isolates were less likely to be multidrug-resistant (8.8% of MSSA and 7.6% of MSCoNS isolates).

### In vitro resistance rates by patient age

**Fig 3:**
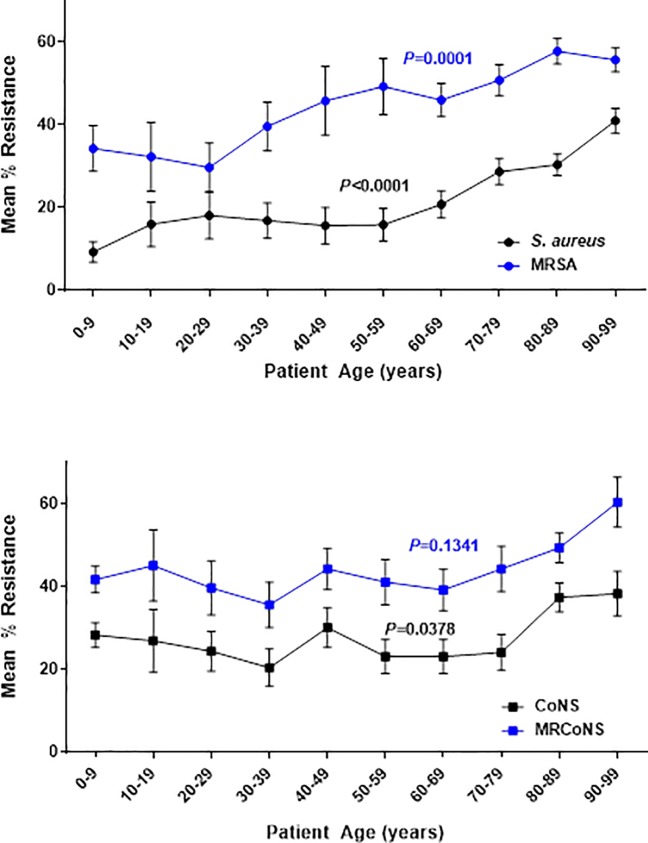
Resistance among staphylococcal isolates from the conjunctiva by patient age. Data are expressed by decade of life for (A) mean ± SE percentage of resistance for *S*. *aureus* (black circles) and MRSA (blue circles); (B) mean ± SE percentage of resistance for CoNS (black squares) and MRCoNS (blue squares). *P*-values are from ANOVAs. Abbreviations: MRSA, methicillin-resistant *S*. *aureus*; CoNS, coagulase-negative staphylococci; MRCoNS, methicillin-resistant coagulase-negative staphylococci.

As shown in **Fig 3**, there was a general pattern of increasing mean percentage in vitro resistance with increasing age by decade of life among both *S*. *aureus* (*P*<0.0001) and CoNS (*P* = 0.0378), as well as for MRSA (*P* = 0.0001). For *S*. *aureus* isolates, pairwise differences were found between isolates from patients aged 0–9 years compared to those aged ≥70 years, those aged 50–59 years compared to those aged ≥80, and for all patients <90 compared to those ≥90. For CoNS isolates, pairwise differences were found between those from patients aged 30–39 years when compared to those from patients aged 80–89 years. Further, among the subset of MRSA isolates specifically, pairwise differences in mean percentage of resistance were found between isolates from patients aged 0–9 years compared to those aged ≥80 years, and between isolates from patients aged 20–29 years compared to those aged ≥70 years. There was no evidence of an association between age by decade of life and mean percentage of resistance among MRCoNS (*P* = 0.1341), *S*. *pneumoniae* (*P* = 0.1760), *P*. *aeruginosa* (*P* = 0.5308), or *H*. *influenzae* (*P =* 0.9846).

**Fig 4:**
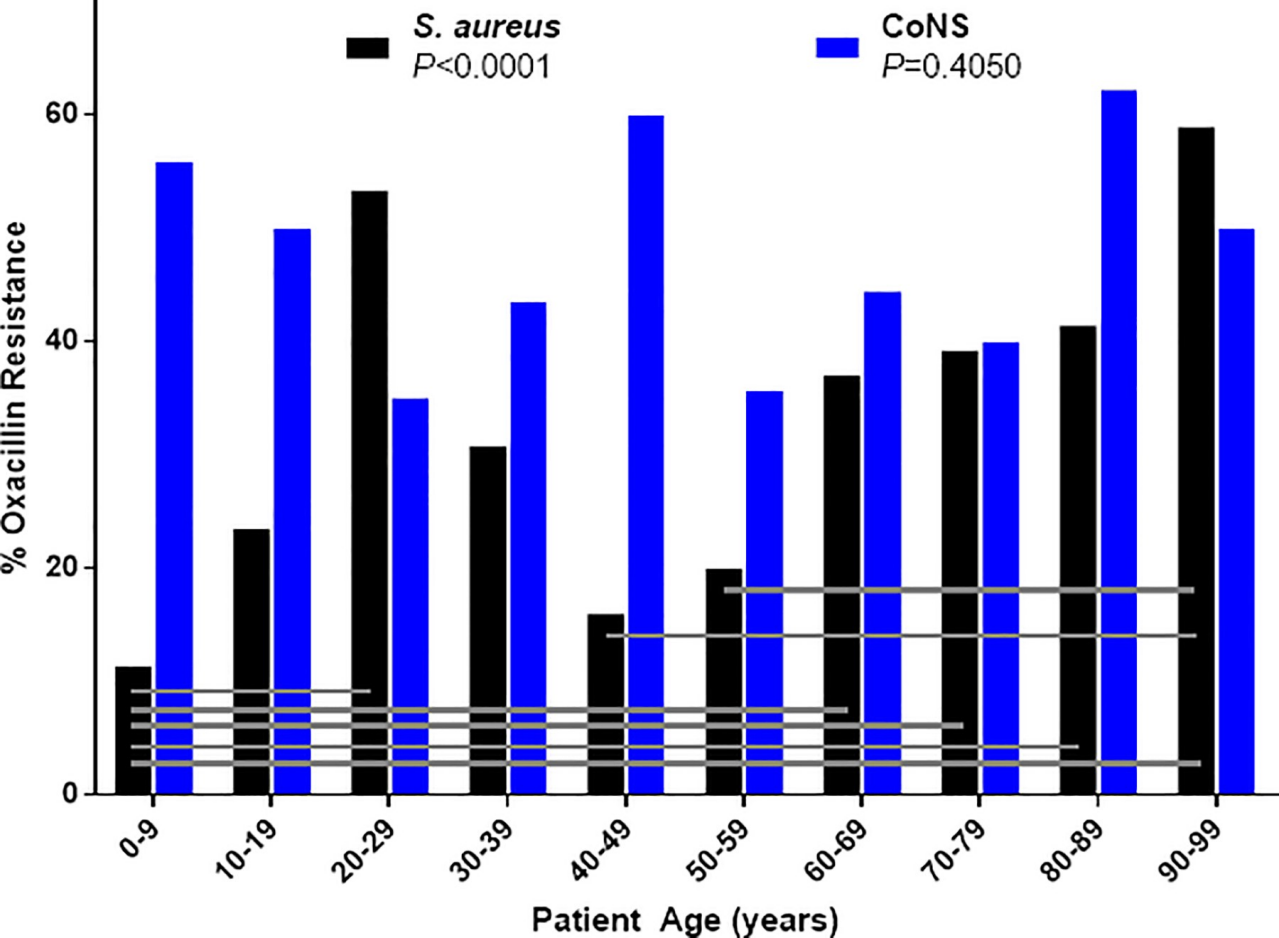
Oxacillin resistance among conjunctival isolates of staphylococci by patient age. Data are expressed by decade of life for percentage of oxacillin resistance among *S*. *aureus* and CoNS; *P*-values are from Chi-Square test. Horizontal lines represent significant pairwise differences (*P*<0.05). Abbreviations: CoNS, coagulase-negative staphylococci.

In vitro oxacillin resistance (**Fig 4**) also differed by age group for *S*. *aureus* (*P*<0.0001), with lower resistance observed among isolates from patients aged 0–9 years compared to those aged 20–29 and ≥60 years, and higher resistance in isolates from patients aged ≥90 years compared to those aged 40–59 years; no differences in oxacillin resistance by age were observed for CoNS (*P* = 0.4050).

### In vitro resistance rates by geography

When analyzing in vitro resistance rates for isolates by geographic region of origin, significant differences were found across regions in mean percentage of resistance for *S*. *aureus* (*P* = 0.0002) and *S*. *pneumoniae* (*P* = 0.0003). Mean (standard error, SE) *S*. *aureus* resistance was highest in the Southern (30.4% [2.6]) and Northeastern (24.1% [2.2) regions and lowest in the Midwestern (18.6% [1.7]) and Western (17.0% [2.1]) regions. Mean (SE) percentage of resistance in *S*. *pneumoniae* was highest in the Midwestern region (31.2% [3.4]) and lowest in the Northeastern (14.3% [5.0]), Western (9.5% [4.6]), and Southern (6.5% [8.3]) regions. There was no evidence for regional differences among mean percentage of resistance for MRSA, CoNS, MRCoNS, *P*. *aeruginosa*, or *H*. *influenzae*.

Similarly, a significant difference was noted with respect to oxacillin resistance specifically between regions for *S*. *aureus* (*P =* 0.0002). *S*. *aureus* resistance to oxacillin was 22.2%, 26.6%, 38.1%, and 48.7% in the Western, Midwestern, Northeastern, and Southern regions respectively, with significance differences between the Midwestern and Southern regions, and between the Western and both Northeastern and Southern regions. There was no evidence of differences across regions regarding resistance to oxacillin for CoNS.

### In vitro resistance rates over time

**Fig 5:**
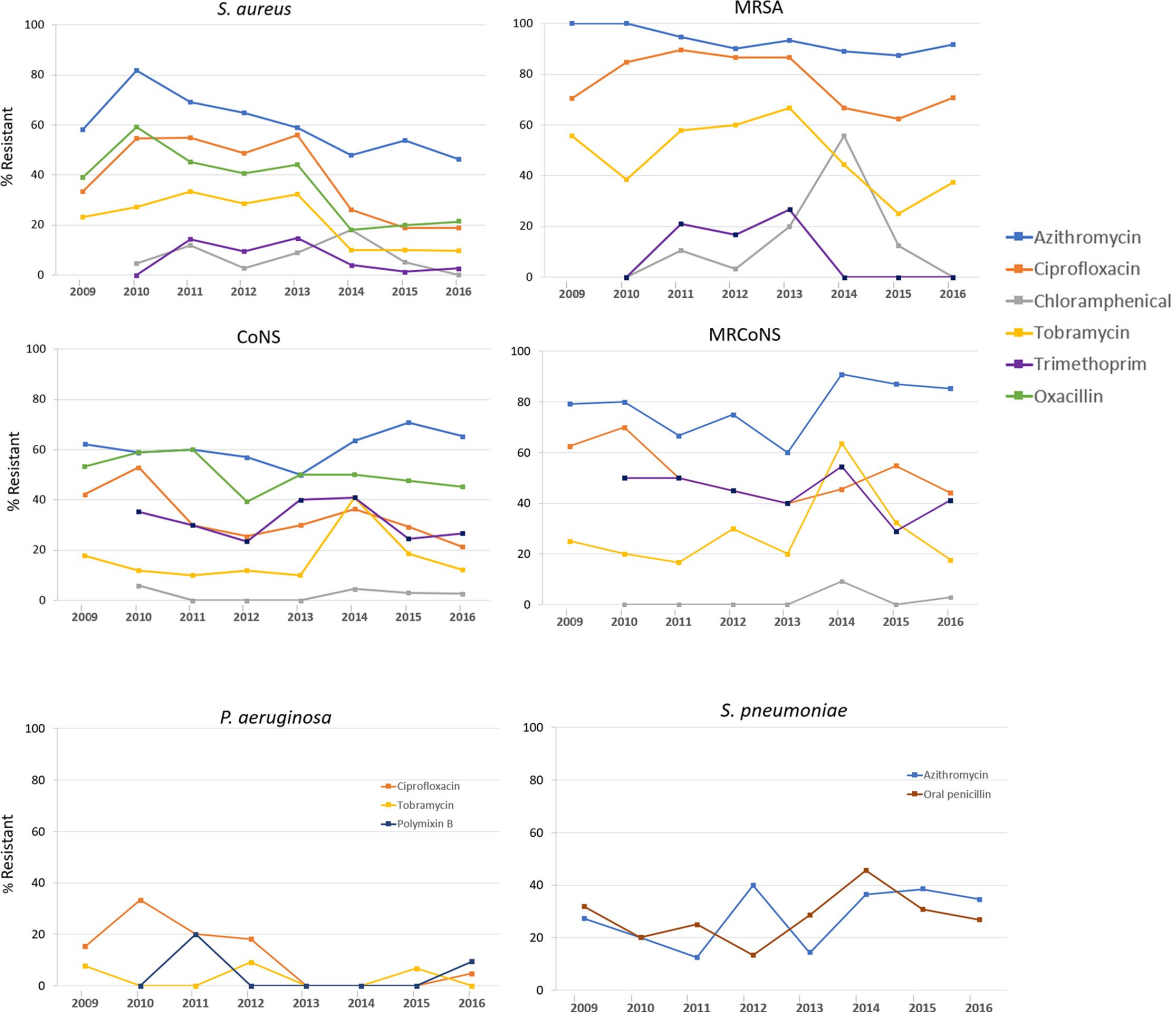
Resistance among ocular *S*. *aureus*, coagulase-negative staphylococci, *P*. *aeruginosa*, and *S*. *pneumoniae* from patients with presumed conjunctivitis by antibiotic class over the 8-year study period. Abbreviations: CoNS, coagulase-negative staphylococci; MRCoNS, methicillin-resistant coagulase-negative staphylococci; MRSA, methicillin-resistant *S*. *aureus*.

Over the eight-year study period, there were small yet significant decreases in in vitro resistance rates for *S*. *aureus* to azithromycin (*P* = 0.0028), ciprofloxacin (*P*<0.0001), tobramycin (*P* = 0.0001), trimethoprim (*P* = 0.0077), and oxacillin (*P*<0.0001) (**Fig 5**). For MRSA isolates, a decrease in resistance from 2009 to 2016 was found only to trimethoprim (*P* = 0.0478). There were also small yet significant decreases in in vitro resistance to ciprofloxacin among CoNS (*P* = 0.0125) and *P*. *aeruginosa* (*P* = 0.0249) isolates. Oxacillin resistance did not change among CoNS (*P* = 0.3298), nor were there significant changes in resistance rates for MRCoNS or *S*. *pneumoniae* to any of the tested antibiotics over the eight-year study period.

## Discussion

Initiated in 2009, the ARMOR study is the only prospective, ongoing, multicenter, nationwide surveillance study designed to monitor antibiotic resistance among *S*. *aureus*, CoNS, *S*. *pneumoniae*, *H*. *influenzae*, and *P*. *aeruginosa* isolates from ocular infections. The current analysis of almost 1200 isolates obtained from 2009 through 2016 sourced from the conjunctiva and presumed causative in bacterial conjunctivitis is the largest dataset on such isolates to our knowledge. Findings from the current analysis indicate substantial levels of in vitro resistance to commonly used antibiotics, particularly among staphylococci species, almost half of which demonstrated resistance to oxacillin. Methicillin resistant staphylococci exhibited increased resistance to other classes of antibiotics compared to methicillin sensitive strains—as attested to by the analysis of multidrug resistance and mean percentage of resistance. Conversely, resistance among *S*. *pneumoniae* isolates was notable only for azithromycin and penicillin, while both *P*. *aeruginosa* and *H*. *influenzae* isolates appeared highly susceptible to all tested antibiotics.

While there are little published data on antibiotic resistance among ocular pathogens, and none specific to common conjunctival isolates on a national level, the findings are generally consistent with previously reported single-center or regional studies in the US that evaluated susceptibility patterns of conjunctival isolates. An analysis of 12,134 presumed bacterial conjunctivitis isolates from the northeastern US [4] found patterns of increased in vitro resistance to older as opposed to newer generation fluoroquinolones among *S*. *aureus*, similar to the current ARMOR analysis; 30.1% of *S*. *aureus* isolates in that study were oxacillin-resistant (MRSA) [4]. Other studies that reported resistance data for conjunctival isolates obtained from clinical situations other than conjunctivitis have noted high rates of in vitro oxacillin resistance similar to that observed in the ARMOR data. In a Stanford University (California) study of isolates obtained prior to intravitreal injection, 47% of CoNS isolates were resistant to oxacillin [48]; a similar prevalence of oxacillin resistance (46.6%) was found among CoNS isolates obtained from cataract surgery patients in the midwestern US [49].

Of note, compared to the 7-year ARMOR results [34], which was inclusive of all ocular isolates and not limited to those obtained from the conjunctiva only, there was little variation in overall cumulative in vitro resistance observed. These findings indicate that bacterial resistance rates among the subset of isolates collected specifically from the conjunctiva reflect those observed in the larger and broader ARMOR dataset and suggests antibiotic resistance may not differ much by etiology although additional study is needed. The majority of MIC_90_ patterns also paralleled previous ARMOR reports [15,16,34], with all tested staphylococcal isolates remaining susceptible to vancomycin. Lower MICs were found when testing isolates against newer fluoroquinolones as opposed to older fluoroquinolones, especially among the staphylococci isolates. The only notable difference between the comprehensive 7-year ARMOR data and the current conjunctival data was found among MRSA for trimethoprim (MIC_90_ of 2 vs MIC_90_ >128, respectively). The reason for this difference is unclear but likely due to the relative impact of a small number (10.3%) of MRSA isolates with high in vitro resistance to trimethoprim to the current MRSA data set as opposed to the larger comprehensive 7-year MRSA data set (4.5-fold larger). For gram-positive isolates, the antibiotic with the lowest MIC_90_ in both this study and the 7-year data set [34] was besifloxacin, a chloro-fluoroquinolone developed for topical ophthalmic use only [50–55]. There were no changes over time in besifloxacin MIC_90_s in either data set, attesting to its balanced inhibition of both DNA gyrase and topoisomerase IV, and low in vitro mutation frequency in mutant selection experiments [56].

When analyzing resistance trends by age, an overall increase in antibiotic resistance with greater age was present among staphylococci, consistent with prior reports [15,16,57]. Older patients are more likely than younger patients to be exposed to environments (eg, nursing homes, hospitals) in which close living conditions, coupled with the presence of high levels of antibiotic-resistant pathogens, allow for rapid spread of resistant infections. Analysis by geographic region revealed significant findings only for *S*. *aureus*, in which there was little variation from previous ARMOR findings [15]. In general, mean percentage resistance rates, as well as oxacillin resistance, among *S*. *aureus* isolates appeared to remain high in the South and low in the West. While the reasons for this geographic disparity are unknown, it could be due to differences in regional prescribing patterns and stewardship practices.

Overall, the decreasing trends for in vitro resistance of conjunctival-sourced isolates over the 8-year study period are encouraging. All staphylococcal and *P*. *aeruginosa* isolates demonstrated significant decreases in resistance to ciprofloxacin, and *S*. *aureus* isolates also demonstrated significant decreases in resistance to oxacillin, azithromycin, and tobramycin. These data generally paralleled the comprehensive 7-year analysis trends [34], although the previously reported small increases in resistance to tobramycin among MRCoNS and to azithromycin among *S*. *pneumoniae* were not duplicated in the current analysis. Oxacillin resistance among CoNS did not increase in either study. The noted decreases in resistance to some antibiotics may reflect more judicious antibiotic use and an improved awareness of measures aimed to combat the growing concern of antibiotic resistance.

While the ARMOR surveillance study was not designed to correlate in vitro susceptibility data with clinical treatment, nor does it collect treatment outcome data, associations between in vitro susceptibility and clinical outcomes have been suggested elsewhere. A retrospective, cross-sectional review of pediatric ocular and peri-ocular infection cases (40% conjunctivitis) with culture-positive MRSA isolates in a northern California pediatric population showed high in vitro resistance to multiple antibiotics with many topical treatment failures [26]. Wilhelmus et al [27] prospectively studied the clinical impact of MICs on clinical response of culture-confirmed bacterial keratitis in patients treated with ciprofloxacin; findings indicated a 43% reduction in improvement and 29% reduction in cure rate among cases in which the pathogen MIC for ciprofloxacin was >1 μg/mL compared to infections caused by organisms with greater in vitro sensitivity (ie, lower MIC).

The current study is subject to several limitations. All samples were obtained within the US, thereby limiting the global generalizability of the data. Although community hospitals and reference laboratories were included as participating centers, the majority of isolates in ARMOR are obtained from hospitals or referral centers and resistance rates therefore may not reflect antibiotic resistance rates in community practices where cultures are seldom taken in cases of suspected bacterial conjunctivitis. For this reason and given ARMOR investigators are instructed only to submit isolates for cases deemed clinically significant, there is likely sampling bias towards more severe conjunctivitis cases. Another limitation is the choice of antibiotics tested; alternate antibiotics within a drug class may have been selected for susceptibility testing. As well, systemic breakpoints were used to interpret MIC data for ocular isolates, and for some antibiotics tested (eg, besifloxacin) there were no established breakpoints to help interpret MIC data. The value of systemic breakpoints for topical ocular treatments remains unclear, owing to the unique pharmacokinetic consequences of ocular administration. On one hand, topical application allows for much higher immediate drug concentrations at the infection site than would be achievable with systemic drug administration, potentially leading to over-reporting of resistance when utilizing systemic breakpoints [58]. On the other hand, topically applied antibiotics are subjected to rapid dilution and removal through blinking and tear turnover, phenomena which limit residence time on the surface of the eye. Yet, as mentioned, some studies applying systemic breakpoints in ocular infections have correlated in vitro resistance with treatment failures [26–30]. Thus, in the absence of topical breakpoints, systemic breakpoints remain useful in determining the antibiotic resistance patterns of ocular isolates and relative susceptibilities to various antibiotics.

## Conclusions

Data from the nationwide ARMOR surveillance study indicate that antibiotic in vitro resistance rates did not increase among ocular isolates originating from the conjunctiva collected from 2009 through 2016. Instead, there was a favorable trend of decreased resistance for certain antibiotic/pathogen combinations, including a decrease in oxacillin resistance among *S*. *aureus*. Despite these positive findings, antibiotic resistance and multidrug resistance remain high among conjunctival isolates, particularly among *S*. *aureus* and CoNS pathogens. Due to the likely sampling bias towards more severe cases of bacterial conjunctivitis, these resistance data should be interpreted with caution.

## Supporting information

S1 Text Age analysis. Statistical analyses of resistance among isolates from the conjunctiva by patient age.
(DOCX)

S2 Text Geographic analysis. Statistical analyses of resistance among isolates from the conjunctiva by geographic region.
(DOCX)

S1 Table Longitudinal trend data. Resistance among isolates from the conjunctiva by antibiotic class over the 8-year study period.
(XLS)

S2 Table Multidrug resistance data. Multidrug resistance among staphylococcal isolates from the conjunctiva.
(XLS)
