# Supplementary material for: Antibiotic resistance among bacterial conjunctival pathogens collected in the Antibiotic Resistance Monitoring in Ocular Microorganisms (ARMOR) surveillance study
Source: PLoS One. 2018 Oct 18;13(10):e0205814. doi: 10.1371/journal.pone.0205814 (PMC6193682; doi:10.1371/journal.pone.0205814)
Supplement: S1 Text — Statistical analyses of resistance among isolates from the conjunctiva by patient age. (DOCX) [file pone.0205814.s001.docx]

# CoNS

**One-Way AOV for Pct_IorR by AgeDecade**

**Source DF SS MS F P**

AgeDecade 9 0.8345 0.09272 2.02 **0.0378**

Error 246 11.2969 0.04592

Total **255** 12.1314

Grand Mean 0.2777 CV 77.16

**Homogeneity of Variances F P**

Levene's Test 1.80 0.0686

O'Brien's Test 1.90 0.0522

Brown and Forsythe Test 1.30 0.2355

**Welch's Test for Mean Differences**

**Source DF F P**

AgeDecade 9.0 1.84 0.0752

Error 73.0

Component of variance for between groups 1.869E-03

Effective cell size 25.0

**AgeDecade N Mean SE**

0 52 0.2826 0.0297

1 8 0.2691 0.0758

2 20 0.2437 0.0479

3 23 0.2041 0.0447

4 20 0.3007 0.0479

5 28 0.2307 0.0405

6 27 0.2310 0.0412

7 25 0.2406 0.0429

8 37 0.3739 0.0352

9 16 0.3828 0.0536

**Tukey HSD All-Pairwise Comparisons Test of Pct_IorR by AgeDecade**

**AgeDecade Mean 0 1 2 3 4 5 6**

0 0.2826

1 0.2691 0.0135

2 0.2437 0.0388 0.0253

3 0.2041 0.0785 0.0650 0.0396

4 0.3007 0.0181 0.0316 0.0569 0.0966

5 0.2307 0.0519 0.0384 0.0131 0.0265 0.0700

6 0.2310 0.0516 0.0381 0.0128 0.0269 0.0697 0.0003

7 0.2406 0.0420 0.0285 0.0032 0.0364 0.0601 0.0099 0.0096

8 0.3739 0.0913 0.1048 0.1301 **0.1698*** 0.0732 0.1432 0.1429

9 0.3828 0.1002 0.1137 0.1391 0.1787 0.0821 0.1522 0.1518

**AgeDecade Mean 7 8**

8 0.3739 0.1333

9 0.3828 0.1423 0.0089

Alpha 0.1 Standard Error for Comparison 0.0461 TO 0.0928

Critical Q Value 4.129 Critical Value for Comparison 0.1346 TO 0.2709

**The homogeneous group format can't be used because of the pattern of significant differences.**

# MRCoNS

**One-Way AOV for Pct_IorR by AgeDecade**

**Source DF SS MS F P**

AgeDecade 9 0.41840 0.04649 1.56 **0.1341**

Error 115 3.41670 0.02971

Total **124** 3.83511

Grand Mean 0.4400 CV 39.17

**Homogeneity of Variances F P**

Levene's Test 1.60 0.1229

O'Brien's Test 1.84 0.0678

Brown and Forsythe Test 0.99 0.4560

**Welch's Test for Mean Differences**

**Source DF F P**

AgeDecade 9.0 1.14 0.3641

Error 30.3

Component of variance for between groups 1.394E-03

Effective cell size 12.0

**AgeDecade N Mean SE**

0 29 0.4171 0.0320

1 4 0.4514 0.0862

2 7 0.3968 0.0651

3 10 0.3556 0.0545

4 12 0.4433 0.0498

5 10 0.4111 0.0545

6 12 0.3924 0.0498

7 10 0.4431 0.0545

8 23 0.4940 0.0359

9 8 0.6042 0.0609

# CoNS vs. OXA

**Cross Tabulation of AgeDecade by OXAsus27**

**OXAsus27**

**AgeDecade R S**

┌──────┬──────┐

**0**  │ 29 │ 23 │ 52

**Row %** │ 55.8 │ 44.2 │ 20.3

├──────┼──────┤

**1**  │ 4 │ 4 │ 8

│ 50.0 │ 50.0 │ 3.1

├──────┼──────┤

**2**  │ 7 │ 13 │ 20

│ 35.0 │ 65.0 │ 7.8

├──────┼──────┤

**3**  │ 10 │ 13 │ 23

│ 43.5 │ 56.5 │ 9.0

├──────┼──────┤

**4**  │ 12 │ 8 │ 20

│ 60.0 │ 40.0 │ 7.8

├──────┼──────┤

**5**  │ 10 │ 18 │ 28

│ 35.7 │ 64.3 │ 10.9

├──────┼──────┤

**6**  │ 12 │ 15 │ 27

│ 44.4 │ 55.6 │ 10.5

├──────┼──────┤

**7**  │ 10 │ 15 │ 25

│ 40.0 │ 60.0 │ 9.8

├──────┼──────┤

**8**  │ 23 │ 14 │ 37

│ 62.2 │ 37.8 │ 14.5

├──────┼──────┤

**9**  │ 8 │ 8 │ 16

│ 50.0 │ 50.0 │ 6.3

└──────┴──────┘

125 131 256

Cases Included 256 Missing Cases 49

**Chi-Square Test for Heterogeneity or Independence**

**for 1 = AgeDecade OXAsus27**

**OXAsus27**

**AgeDecade R S**

┌───────┬───────┐

0 Observed │ 29 │ 23 │ 52

Expected │ 25.39 │ 26.61 │

Cell χ² │ 0.51 │ 0.49 │

├───────┼───────┤

1 Observed │ 4 │ 4 │ 8

Expected │ 3.91 │ 4.09 │

Cell χ² │ 0.00 │ 0.00 │

├───────┼───────┤

2 Observed │ 7 │ 13 │ 20

Expected │ 9.77 │ 10.23 │

Cell χ² │ 0.78 │ 0.75 │

├───────┼───────┤

3 Observed │ 10 │ 13 │ 23

Expected │ 11.23 │ 11.77 │

Cell χ² │ 0.13 │ 0.13 │

├───────┼───────┤

4 Observed │ 12 │ 8 │ 20

Expected │ 9.77 │ 10.23 │

Cell χ² │ 0.51 │ 0.49 │

├───────┼───────┤

5 Observed │ 10 │ 18 │ 28

Expected │ 13.67 │ 14.33 │

Cell χ² │ 0.99 │ 0.94 │

├───────┼───────┤

6 Observed │ 12 │ 15 │ 27

Expected │ 13.18 │ 13.82 │

Cell χ² │ 0.11 │ 0.10 │

├───────┼───────┤

7 Observed │ 10 │ 15 │ 25

Expected │ 12.21 │ 12.79 │

Cell χ² │ 0.40 │ 0.38 │

├───────┼───────┤

8 Observed │ 23 │ 14 │ 37

Expected │ 18.07 │ 18.93 │

Cell χ² │ 1.35 │ 1.29 │

├───────┼───────┤

9 Observed │ 8 │ 8 │ 16

Expected │ 7.81 │ 8.19 │

Cell χ² │ 0.00 │ 0.00 │

└───────┴───────┘

125 131 256

Overall Chi-Square 9.36

P-value **0.4050** Degrees of Freedom 9

# Sau

**One-Way AOV for Pct_IorR by AgeDecade**

**Source DF SS MS F P**

AgeDecade 9 4.3789 0.48655 9.98 **0.0000**

Error 405 19.7524 0.04877

Total **414** 24.1313

Grand Mean 0.2271 CV 97.24

**Homogeneity of Variances F P**

Levene's Test 6.22 0.0000

O'Brien's Test 5.89 0.0000

Brown and Forsythe Test 7.31 0.0000

**Welch's Test for Mean Differences**

**Source DF F P**

AgeDecade 9.0 12.28 0.0000

Error 113.4

Component of variance for between groups 0.01086

Effective cell size 40.3

**AgeDecade N Mean SE**

0 79 0.0927 0.0248

1 17 0.1601 0.0536

2 15 0.1815 0.0570

3 26 0.1688 0.0433

4 25 0.1567 0.0442

5 30 0.1588 0.0403

6 46 0.2077 0.0326

7 51 0.2868 0.0309

8 70 0.3036 0.0264

9 56 0.4105 0.0295

**Tukey HSD All-Pairwise Comparisons Test of Pct_IorR by AgeDecade**

**AgeDecade Mean 0 1 2 3 4 5 6**

0 0.0927

1 0.1601 0.0675

2 0.1815 0.0888 0.0214

3 0.1688 0.0762 0.0087 0.0127

4 0.1567 0.0640 0.0035 0.0248 0.0121

5 0.1588 0.0661 0.0013 0.0227 0.0100 0.0021

6 0.2077 0.1151 0.0476 0.0262 0.0389 0.0511 0.0489

7 0.2868 **0.1941*** 0.1266 0.1053 0.1180 0.1301 0.1280 0.0790

8 0.3036 **0.2109*** 0.1434 0.1221 0.1348 0.1469 **0.1448*** 0.0958

9 0.4105 **0.3178*** **0.2503*** **0.2290*** **0.2417*** **0.2538*** **0.2517*** **0.2027***

**AgeDecade Mean 7 8**

8 0.3036 0.0168

9 0.4105 0.1237 0.1069

Alpha 0.1 Standard Error for Comparison 0.0363 TO 0.0782

Critical Q Value 4.129 Critical Value for Comparison 0.1058 TO 0.2284

**The homogeneous group format can't be used because of the pattern of significant differences.**

# Sau, MRSA

**One-Way AOV for Pct_IorR by AgeDecade**

**Source DF SS MS F P**

AgeDecade 9 1.07458 0.11940 4.34 **0.0001**

Error 128 3.52397 0.02753

Total 137 4.59855

Grand Mean 0.4915 CV 33.76

**Homogeneity of Variances F P**

Levene's Test 1.68 0.1000

O'Brien's Test 1.69 0.0972

Brown and Forsythe Test 1.22 0.2901

**Welch's Test for Mean Differences**

**Source DF F P**

AgeDecade 9.0 7.04 0.0000

Error 25.8

Component of variance for between groups 7.066E-03

Effective cell size 13.0

**AgeDecade N Mean SE**

0 9 0.3426 0.0553

1 4 0.3229 0.0830

2 8 0.2969 0.0587

3 8 0.3958 0.0587

4 4 0.4583 0.0830

5 6 0.4931 0.0677

6 17 0.4600 0.0402

7 20 0.5083 0.0371

8 29 0.5781 0.0308

9 33 0.5568 0.0289

**Tukey HSD All-Pairwise Comparisons Test of Pct_IorR by AgeDecade**

**AgeDecade Mean 0 1 2 3 4 5 6**

0 0.3426

1 0.3229 0.0197

2 0.2969 0.0457 0.0260

3 0.3958 0.0532 0.0729 0.0990

4 0.4583 0.1157 0.1354 0.1615 0.0625

5 0.4931 0.1505 0.1701 0.1962 0.0972 0.0347

6 0.4600 0.1174 0.1371 0.1631 0.0641 0.0016 0.0331

7 0.5083 0.1657 0.1854 **0.2115*** 0.1125 0.0500 0.0153 0.0484

8 0.5781 **0.2355*** 0.2551 **0.2812*** 0.1822 0.1197 0.0850 0.1181

9 0.5568 **0.2142*** 0.2339 **0.2599*** 0.1610 0.0985 0.0638 0.0969

**AgeDecade Mean 7 8**

8 0.5781 0.0697

9 0.5568 0.0485 0.0212

Alpha 0.1 Standard Error for Comparison 0.0422 TO 0.1173

Critical Q Value 4.129 Critical Value for Comparison 0.1233 TO 0.3426

**The homogeneous group format can't be used because of the pattern of significant differences.**

# Sau vs. OXA

**Cross Tabulation of AgeDecade by OXAsus27**

**OXAsus27**

**AgeDecade R S**

┌──────┬──────┐

**0**  │ 9 │ 70 │ 79

**Row %** │ 11.4 │ 88.6 │ 19.0

├──────┼──────┤

**1**  │ 4 │ 13 │ 17

│ 23.5 │ 76.5 │ 4.1

├──────┼──────┤

**2**  │ 8 │ 7 │ 15

│ 53.3 │ 46.7 │ 3.6

├──────┼──────┤

**3**  │ 8 │ 18 │ 26

│ 30.8 │ 69.2 │ 6.3

├──────┼──────┤

**4**  │ 4 │ 21 │ 25

│ 16.0 │ 84.0 │ 6.0

├──────┼──────┤

**5**  │ 6 │ 24 │ 30

│ 20.0 │ 80.0 │ 7.2

├──────┼──────┤

**6**  │ 17 │ 29 │ 46

│ 37.0 │ 63.0 │ 11.1

├──────┼──────┤

**7**  │ 20 │ 31 │ 51

│ 39.2 │ 60.8 │ 12.3

├──────┼──────┤

**8**  │ 29 │ 41 │ 70

│ 41.4 │ 58.6 │ 16.9

├──────┼──────┤

**9**  │ 33 │ 23 │ 56

│ 58.9 │ 41.1 │ 13.5

└──────┴──────┘

138 277 415

Cases Included 415 Missing Cases 68

**Chi-Square Test for Heterogeneity or Independence**

**for 1 = AgeDecade OXAsus27**

**OXAsus27**

**AgeDecade R S**

┌───────┬───────┐

0 Observed │ 9 │ 70 │ 79

Expected │ 26.27 │ 52.73 │

Cell χ² │ 11.35 │ 5.66 │

├───────┼───────┤

1 Observed │ 4 │ 13 │ 17

Expected │ 5.65 │ 11.35 │

Cell χ² │ 0.48 │ 0.24 │

├───────┼───────┤

2 Observed │ 8 │ 7 │ 15

Expected │ 4.99 │ 10.01 │

Cell χ² │ 1.82 │ 0.91 │

├───────┼───────┤

3 Observed │ 8 │ 18 │ 26

Expected │ 8.65 │ 17.35 │

Cell χ² │ 0.05 │ 0.02 │

├───────┼───────┤

4 Observed │ 4 │ 21 │ 25

Expected │ 8.31 │ 16.69 │

Cell χ² │ 2.24 │ 1.11 │

├───────┼───────┤

5 Observed │ 6 │ 24 │ 30

Expected │ 9.98 │ 20.02 │

Cell χ² │ 1.58 │ 0.79 │

├───────┼───────┤

6 Observed │ 17 │ 29 │ 46

Expected │ 15.30 │ 30.70 │

Cell χ² │ 0.19 │ 0.09 │

├───────┼───────┤

7 Observed │ 20 │ 31 │ 51

Expected │ 16.96 │ 34.04 │

Cell χ² │ 0.55 │ 0.27 │

├───────┼───────┤

8 Observed │ 29 │ 41 │ 70

Expected │ 23.28 │ 46.72 │

Cell χ² │ 1.41 │ 0.70 │

├───────┼───────┤

9 Observed │ 33 │ 23 │ 56

Expected │ 18.62 │ 37.38 │

Cell χ² │ 11.10 │ 5.53 │

└───────┴───────┘

138 277 415

Overall Chi-Square 46.10

P-value **0.0000** Degrees of Freedom 9

**Multiple Comparisons for Proportions**

**AgeDecade P Angle(P) 0 1 2 3 4 5**

0 0.1139 20.15

1 0.2353 29.97 9.81

2 0.5333 46.80 **26.64*** 16.83

3 0.3077 34.12 13.97 4.16 12.67

4 0.1600 24.55 4.40 5.41 22.24 9.57

5 0.2000 27.24 7.08 2.73 19.56 6.89 2.68

6 0.3696 37.60 **17.45*** 7.64 9.19 3.48 13.05 10.37

7 0.3922 38.89 **18.74*** 8.93 7.90 4.77 14.34 11.66

8 0.4143 40.13 **19.98*** 10.17 6.66 6.01 15.58 12.90

9 0.5893 50.05 **29.90*** 20.09 3.26 15.93 **25.50*** **22.82***

**AgeDecade P Angle(P) 6 7 8**

7 0.3922 38.89 1.29

8 0.4143 40.13 2.53 1.24

9 0.5893 50.05 12.45 11.16 9.92

Alpha 0.05

Critical Q Value 4.474

# Hin

**One-Way AOV for Pct_IorR by AgeDecade**

**Source DF SS MS F P**

AgeDecade 9 0.00733 8.147E-04 0.26 **0.9846**

Error 163 0.51579 3.164E-03

Total **172** 0.52312

Grand Mean 8.19E-03 CV 686.94

**Homogeneity of Variances F P**

Levene's Test 0.23 0.9903

O'Brien's Test M M

Brown and Forsythe Test 0.26 0.9846

**Welch's Test for Mean Differences**

**Source DF F P**

AgeDecade 8.0 M M

Error M

Component of variance for between groups -2.025E-04

Effective cell size 11.6

**AgeDecade N Mean SE**

0 106 0.0134 0.0055

1 1 0.0000 0.0563

2 5 0.0000 0.0252

3 5 0.0000 0.0252

4 4 0.0000 0.0281

5 9 0.0000 0.0188

6 14 0.0000 0.0150

7 10 0.0000 0.0178

8 9 0.0000 0.0188

9 10 0.0000 0.0178

# Pae

**One-Way AOV for Pct_IorR by AgeDecade**

**Source DF SS MS F P**

AgeDecade 9 0.26662 0.02962 0.90 **0.5308**

Error 58 1.90781 0.03289

Total **67** 2.17443

Grand Mean 0.0662 CV 274.06

**Homogeneity of Variances F P**

Levene's Test 1.23 0.2929

O'Brien's Test M M

Brown and Forsythe Test 0.90 0.5308

**Welch's Test for Mean Differences**

**Source DF F P**

AgeDecade 8.0 M M

Error M

Component of variance for between groups -4.924E-04

Effective cell size 6.6

**AgeDecade N Mean SE**

0 7 0.0000 0.0685

1 2 0.0000 0.1282

2 7 0.0476 0.0685

3 6 0.0000 0.0740

4 6 0.0556 0.0740

5 8 0.0833 0.0641

6 9 0.2037 0.0605

7 11 0.0909 0.0547

8 11 0.0303 0.0547

9 1 0.0000 0.1814

# Spn

**One-Way AOV for Pct_IorR by AgeDecade**

**Source DF SS MS F P**

AgeDecade 9 0.92051 0.10228 1.46 **0.1760**

Error 89 6.24209 0.07014

Total **98** 7.16260

Grand Mean 0.1987 CV 133.31

**Homogeneity of Variances F P**

Levene's Test 1.65 0.1139

O'Brien's Test M M

Brown and Forsythe Test 1.42 0.1923

**Welch's Test for Mean Differences**

**Source DF F P**

AgeDecade 9.0 M M

Error M

Component of variance for between groups 4.145E-03

Effective cell size 7.8

**AgeDecade N Mean SE**

0 51 0.2435 0.0371

1 5 0.0667 0.1184

2 6 0.2083 0.1081

3 7 0.0833 0.1001

4 8 0.0938 0.0936

5 8 0.0417 0.0936

6 4 0.3333 0.1324

7 4 0.2500 0.1324

8 4 0.4167 0.1324

9 2 0.0000 0.1873
