# Supplementary material for: Antibiotic resistance among bacterial conjunctival pathogens collected in the Antibiotic Resistance Monitoring in Ocular Microorganisms (ARMOR) surveillance study
Source: PLoS One. 2018 Oct 18;13(10):e0205814. doi: 10.1371/journal.pone.0205814 (PMC6193682; doi:10.1371/journal.pone.0205814)
Supplement: S2 Text — Statistical analyses of resistance among isolates from the conjunctiva by geographic region. (DOCX) [file pone.0205814.s002.docx]

# CoNS

**One-Way AOV for Pct_IorR by Region**

**Source DF SS MS F P**

Region 3 0.0814 0.02715 0.60 **0.6173**

Error 301 13.6849 0.04546

Total **304** 13.7663

Grand Mean 0.2749 CV 77.57

**Homogeneity of Variances F P**

Levene's Test 1.26 0.2876

O'Brien's Test 1.27 0.2842

Brown and Forsythe Test 1.48 0.2189

**Welch's Test for Mean Differences**

**Source DF F P**

Region 3.0 0.67 0.5742

Error 50.2

Component of variance for between groups -2.632E-04

Effective cell size 69.6

**Region N Mean SE**

Midwestern 113 0.2801 0.0201

Northeastern 101 0.2867 0.0212

Southern 12 0.3009 0.0616

Western 79 0.2482 0.0240

# MRCoNS

**One-Way AOV for Pct_IorR by Region**

**Source DF SS MS F P**

Region 3 0.09187 0.03062 1.00 **0.3948**

Error 143 4.37855 0.03062

Total **146** 4.47042

Grand Mean 0.4292 CV 40.77

**Homogeneity of Variances F P**

Levene's Test 1.09 0.3574

O'Brien's Test 0.95 0.4169

Brown and Forsythe Test 1.16 0.3273

**Welch's Test for Mean Differences**

**Source DF F P**

Region 3.0 1.44 0.2576

Error 21.5

Component of variance for between groups 1.462E-07

Effective cell size 32.6

**Region N Mean SE**

Midwestern 64 0.4191 0.0219

Northeastern 45 0.4586 0.0261

Southern 5 0.4889 0.0783

Western 33 0.3998 0.0305

# CoNS vs. OXA

**Cross Tabulation of Region by OXAsus**

**OXAsus**

**Region R S**

┌──────┬──────┐

**Midwestern**  │ 64 │ 49 │ 113

**Row %** │ 56.6 │ 43.4 │ 37.0

├──────┼──────┤

**Northeastern** │ 45 │ 56 │ 101

│ 44.6 │ 55.4 │ 33.1

├──────┼──────┤

**Southern**  │ 5 │ 7 │ 12

│ 41.7 │ 58.3 │ 3.9

├──────┼──────┤

**Western**  │ 33 │ 46 │ 79

│ 41.8 │ 58.2 │ 25.9

└──────┴──────┘

147 158 305

Cases Included 305 Missing Cases 0

**Chi-Square Test for Heterogeneity or Independence**

**for 1 = Region OXAsus**

**OXAsus**

**Region R S**

┌───────┬───────┐

Midwestern Observed │ 64 │ 49 │ 113

Expected │ 54.46 │ 58.54 │

Cell χ² │ 1.67 │ 1.55 │

├───────┼───────┤

Northeastern Observed │ 45 │ 56 │ 101

Expected │ 48.68 │ 52.32 │

Cell χ² │ 0.28 │ 0.26 │

├───────┼───────┤

Southern Observed │ 5 │ 7 │ 12

Expected │ 5.78 │ 6.22 │

Cell χ² │ 0.11 │ 0.10 │

├───────┼───────┤

Western Observed │ 33 │ 46 │ 79

Expected │ 38.08 │ 40.92 │

Cell χ² │ 0.68 │ 0.63 │

└───────┴───────┘

147 158 305

Overall Chi-Square 5.27

P-value **0.1529**

Degrees of Freedom 3

# Sau

**One-Way AOV for Pct_IorR by Region**

**Source DF SS MS F P**

Region 3 1.0700 0.35666 6.79 **0.0002**

Error 479 25.1735 0.05255

Total **482** 26.2435

Grand Mean 0.2134 CV 107.41

**Homogeneity of Variances F P**

Levene's Test 1.20 0.3088

O'Brien's Test 1.25 0.2911

Brown and Forsythe Test 2.33 0.0731

**Welch's Test for Mean Differences**

**Source DF F P**

Region 3.0 6.01 0.0006

Error 222.2

Component of variance for between groups 2.596E-03

Effective cell size 117.1

**Region N Mean SE**

Midwestern 177 0.1857 0.0172

Northeastern 113 0.2413 0.0216

Southern 76 0.3039 0.0263

Western 117 0.1696 0.0212

**Tukey HSD All-Pairwise Comparisons Test of Pct_IorR by Region**

**Region Mean Homogeneous Groups**

Southern 0.3039 A

Northeastern 0.2413 AB

Midwestern 0.1857 BC

Western 0.1696 C

Alpha 0.1 Standard Error for Comparison 0.0273 TO 0.0340

Critical Q Value 3.240 Critical Value for Comparison 0.0626 TO 0.0779

**There are 3 groups (A, B, etc.) in which the means are not significantly different from one another.**

# Sau, MRSA

**One-Way AOV for Pct_IorR by Region**

**Source DF SS MS F P**

Region 3 0.10013 0.03338 1.01 **0.3921**

Error 149 4.94544 0.03319

Total **152** 5.04557

Grand Mean 0.4792 CV 38.02

**Homogeneity of Variances F P**

Levene's Test 1.39 0.2473

O'Brien's Test 1.35 0.2615

Brown and Forsythe Test 1.48 0.2220

**Welch's Test for Mean Differences**

**Source DF F P**

Region 3.0 1.02 0.3869

Error 75.3

Component of variance for between groups 4.937E-06

Effective cell size 37.7

**Region N Mean SE**

Midwestern 47 0.4477 0.0266

Northeastern 43 0.4735 0.0278

Southern 37 0.5139 0.0300

Western 26 0.4963 0.0357

# Sau vs. OXA

**Cross Tabulation of Region by OXAsus**

**OXAsus**

**Region R S**

┌──────┬──────┐

**Midwestern**  │ 47 │ 130 │ 177

**Row %** │ 26.6 │ 73.4 │ 36.6

├──────┼──────┤

**Northeastern** │ 43 │ 70 │ 113

│ 38.1 │ 61.9 │ 23.4

├──────┼──────┤

**Southern**  │ 37 │ 39 │ 76

│ 48.7 │ 51.3 │ 15.7

├──────┼──────┤

**Western**  │ 26 │ 91 │ 117

│ 22.2 │ 77.8 │ 24.2

└──────┴──────┘

153 330 483

Cases Included 483 Missing Cases 0

**Chi-Square Test for Heterogeneity or Independence**

**for 1 = Region OXAsus**

**OXAsus**

**Region R S**

┌────────┬────────┐

Midwestern Observed │ 47 │ 130 │ 177

Expected │ 56.07 │ 120.93 │

Cell χ² │ 1.47 │ 0.68 │

├────────┼────────┤

Northeastern Observed │ 43 │ 70 │ 113

Expected │ 35.80 │ 77.20 │

Cell χ² │ 1.45 │ 0.67 │

├────────┼────────┤

Southern Observed │ 37 │ 39 │ 76

Expected │ 24.07 │ 51.93 │

Cell χ² │ 6.94 │ 3.22 │

├────────┼────────┤

Western Observed │ 26 │ 91 │ 117

Expected │ 37.06 │ 79.94 │

Cell χ² │ 3.30 │ 1.53 │

└────────┴────────┘

153 330 483

Overall Chi-Square 19.26

P-value **0.0002**

Degrees of Freedom 3

**Multiple Comparisons for Proportions**

**Region P Angle(P) Midwestern Northeastern Southern**

Midwestern 0.2655 31.10

Northeastern 0.3805 38.15 7.05

Southern 0.4868 44.26 **13.15*** 6.11

Western 0.2222 28.29 2.82 **9.86*** **15.97***

Alpha 0.05

Critical Q Value 3.633

# Hin

**One-Way AOV for Pct_IorR by Region**

**Source DF SS MS F P**

Region 3 0.00827 2.757E-03 1.09 **0.3551**

Error 204 0.51680 2.533E-03

Total **207** 0.52507

Grand Mean 6.81E-03 CV 739.00

**Homogeneity of Variances F P**

Levene's Test 0.96 0.4149

O'Brien's Test 0.95 0.4191

Brown and Forsythe Test 1.09 0.3551

**Welch's Test for Mean Differences**

**Source DF F P**

Region 3.0 M M

Error M

Component of variance for between groups 5.062E-06

Effective cell size 44.1

**Region N Mean SE**

Midwestern 112 0.0126 4.76E-03

Northeastern 30 0.0000 9.19E-03

Southern 26 0.0000 9.87E-03

Western 40 0.0000 7.96E-03

# Pae

**One-Way AOV for Pct_IorR by Region**

**Source DF SS MS F P**

Region 3 0.02647 0.00882 0.31 **0.8182**

Error 80 2.27875 0.02848

Total **83** 2.30522

Grand Mean 0.0575 CV 293.32

**Homogeneity of Variances F P**

Levene's Test 0.99 0.4020

O'Brien's Test 0.95 0.4200

Brown and Forsythe Test 0.31 0.8182

**Welch's Test for Mean Differences**

**Source DF F P**

Region 3.0 0.28 0.8383

Error 29.4

Component of variance for between groups -1.059E-03

Effective cell size 18.6

**Region N Mean SE**

Midwestern 42 0.0595 0.0260

Northeastern 17 0.0392 0.0409

Southern 10 0.0333 0.0534

Western 15 0.0889 0.0436

# Spn

**One-Way AOV for Pct_IorR by Region**

**Source DF SS MS F P**

Region 3 1.25496 0.41832 6.70 **0.0003**

Error 114 7.11445 0.06241

Total **117** 8.36941

Grand Mean 0.2041 CV 122.40

**Homogeneity of Variances F P**

Levene's Test 5.67 0.0012

O'Brien's Test 5.13 0.0023

Brown and Forsythe Test 7.86 0.0001

**Welch's Test for Mean Differences**

**Source DF F P**

Region 3.0 7.28 0.0006

Error 38.6

Component of variance for between groups 0.01347

Effective cell size 26.4

**Region N Mean SE**

Midwestern 55 0.3121 0.0337

Northeastern 25 0.1433 0.0500

Southern 9 0.0648 0.0833

Western 29 0.0948 0.0464

**Tukey HSD All-Pairwise Comparisons Test of Pct_IorR by Region**

**Region Mean Homogeneous Groups**

Midwestern 0.3121 A

Northeastern 0.1433 B

Western 0.0948 B

Southern 0.0648 B

Alpha 0.1 Standard Error for Comparison 0.0573 TO 0.0971

Critical Q Value 3.278 Critical Value for Comparison 0.1329 TO 0.2251

**There are 2 groups (A and B) in which the means are not significantly different from one another.**
